# The Role of the Antioxidant Response in Mitochondrial Dysfunction in Degenerative Diseases: Cross-Talk between Antioxidant Defense, Autophagy, and Apoptosis

**DOI:** 10.1155/2019/6392763

**Published:** 2019-04-07

**Authors:** Michael L.-H. Huang, Shannon Chiang, Danuta S. Kalinowski, Dong-Hun Bae, Sumit Sahni, Des R. Richardson

**Affiliations:** Molecular Pharmacology and Pathology Program, Department of Pathology and Bosch Institute, Medical Foundation Building (K25), University of Sydney, Sydney, New South Wales 2006, Australia

## Abstract

The mitochondrion is an essential organelle important for the generation of ATP for cellular function. This is especially critical for cells with high energy demands, such as neurons for signal transmission and cardiomyocytes for the continuous mechanical work of the heart. However, deleterious reactive oxygen species are generated as a result of mitochondrial electron transport, requiring a rigorous activation of antioxidative defense in order to maintain homeostatic mitochondrial function. Indeed, recent studies have demonstrated that the dysregulation of antioxidant response leads to mitochondrial dysfunction in human degenerative diseases affecting the nervous system and the heart. In this review, we outline and discuss the mitochondrial and oxidative stress factors causing degenerative diseases, such as Alzheimer's disease, Parkinson's disease, amyotrophic lateral sclerosis, Huntington's disease, and Friedreich's ataxia. In particular, the pathological involvement of mitochondrial dysfunction in relation to oxidative stress, energy metabolism, mitochondrial dynamics, and cell death will be explored. Understanding the pathology and the development of these diseases has highlighted novel regulators in the homeostatic maintenance of mitochondria. Importantly, this offers potential therapeutic targets in the development of future treatments for these degenerative diseases.

## 1. Mitochondria and Oxidative Stress

Mitochondria are the major energy-producing organelle of the cell *via* the process of oxidative phosphorylation (OXPHOS). In addition to this important role, mitochondria are also involved in a myriad of biological functions, from the generation of vital cellular metabolites such as iron-sulfur clusters (ISCs) and heme [1] to the regulation of cell death [2, 3]. However, as a consequence of active oxidative metabolism, in particular complex I and III of the electron transport chain, mitochondria are also a major source of reactive oxygen species (ROS) in cells [3, 4], with superoxide anions, hydroxyl radicals, and hydrogen peroxide being the predominant forms of ROS [5]. Apart from its well-known role in cytotoxicity, the generation of ROS has important signaling functions, with their levels being regulated by a suite of cellular antioxidants [2].

When the rate of ROS production exceeds cellular antioxidant capacity, the ensuing oxidative stress damages vital components of the cell, resulting in oxidation of membranes, proteins, and nucleic acids. Within the mitochondrion, ROS can potentiate profound damage to mitochondrial energy production by causing mitochondrial DNA (mtDNA) damage and subsequent defects in mtDNA-encoded subunits of the respiratory complex I and III [6]. Furthermore, ROS can readily interact with ISCs within subunits of complex I, II, and III to disrupt their function [6]. The exquisite dependence of neurons and cardiomyocytes on mitochondria for ATP production also means these cells are particularly susceptible to mitochondrial ROS [4, 7]. As such, the accumulation of oxidative damage within cells leads to death and is a driver of aging as well as neurodegenerative and cardiodegenerative diseases [3, 8].

## 2. Mitochondrial DNA

The mtDNA encodes 22 transfer RNAs, two ribosomal RNAs, and 13 essential proteins of oxidative phosphorylation, the quintessential machinery responsible for ATP production [9]. Due to limited mtDNA repair enzymes, absence of protective histone molecules, and the susceptibility of mtDNA to oxidative damage, mtDNA is prone to mutations, which drives further mitochondrial dysfunction and potentiates a vicious cycle of mtDNA damage [4, 7, 10]. Mutations in mtDNA also accumulate with aging [11] or are inherited in a number of human mitochondrial diseases [12]. The importance of maintaining mtDNA integrity in age-related diseases is demonstrated by mice that carry a mutation in the mtDNA polymerase-*γ* (*Polg*), which disables the mtDNA proofreading activity of the enzyme [10]. As a result, Polg mutant mice accumulate mtDNA mutations during mtDNA replication [10] and carry an average of 9 point mutations per 10 kb in cytochrome *b*, versus 1 mutation per 10 kb in control mice [10]. The mutant mice develop pathologies associated with aging, including weight loss, osteoporosis, kyphosis, alopecia, cardiomyopathy, anemia, and sarcopenia [10].

## 3. Mitochondria and Antioxidant Defense

As the mitochondrion is an active site of cellular redox homeostasis and a major source of ROS, it is not surprising that the homeostasis of this organelle can be regulated by the master regulator of cellular antioxidant defense, nuclear factor erythroid-derived 2-related factor 2 [13, 14]. The nuclear factor erythroid-derived 2-related factor 2 is commonly known as NRF2 [13, 14]. However, in order to distinguish it from *Nuclear Respiratory Factor 2* that is involved in regulating mitochondrial biogenesis and bioenergetics, it will be referred to by its gene name, *NFE2L2*. Significantly, NFE2L2 is a well-known transcription factor and a master regulator of a variety of antioxidant and detoxifying enzymes [15]. NFE2L2 heterodimerizes with small musculoaponeurotic fibrosarcoma (sMAF) proteins to enable specific binding to its target DNA sequence known as the antioxidant response element (ARE) [15, 16]. The binding of NFE2L2 to ARE leads to the transcriptional activation of ARE-containing genes; these include major phase II detoxifying enzymes and enzymes in the glutathione, thioredoxin, and peroxiredoxin antioxidant systems (reviewed in [17]).

The expression of NFE2L2 is tightly regulated *via* the proteasomal system [17]. The best known mechanism of NFE2L2 regulation is mediated through the Kelch-like ECH-associated protein 1 (KEAP1) which is the substrate adapter protein for the Cul3-RBX1 E3 ubiquitin ligase complex, which responds to electrophilic and/or oxidative signals [17]. In addition, there is a KEAP1-independent mechanism of NFE2L2 regulation involving glycogen synthase kinase-3*β* (GSK3*β*) that likely responds to receptor-mediated signal transduction [17]. This mechanism involves phosphorylation of nuclear NFE2L2 by GSK3*β*, leading to the recruitment of another E3-ubiquitin ligase adapter, *β*-TrCP [18, 19], or *via* the Src kinase, the Fyn-mediated nuclear NFE2L2 export process [20, 21].

An additional mechanism of NFE2L2 activation involves p62-dependent autophagic degradation of KEAP1 [22–25]. This process could involve the competitive binding of p62, which is reportedly induced by NFE2L2 activity [23], to the NFE2L2-binding site on KEAP1, thereby preventing KEAP1-mediated NFE2L2 degradation [23–25]. Therefore, increased phosphorylated p62-mediated autophagy increases NFE2L2 activity, which in turn increases p62 activity [23, 26].

In addition, NFE2L2 has been shown to directly affect mitochondrial homeostasis via its regulation of nuclear respiratory factor 1 (NRF1) through the 4 AREs in the *NRF1* promoter and thereby promote mitochondrial biogenesis [14]. Other studies have also demonstrated that NFE2L2 is also able to indirectly activate another major driver of mitochondrial biogenesis, the peroxisome proliferator-activated receptor *γ* coactivator-1*α* (PGC1*α*) *via* heme oxygenase-1 (HO-1)/carbon monoxide signaling [13, 27]. As such, NFE2L2 is essential for mitochondrial function, with regulation of NFE2L2 expression strongly and positively modulating mitochondrial membrane potential, ATP production, and efficiency of oxidative phosphorylation [15, 28].

Recently, NFE2L2 has also been identified to be associated with mitochondria through a complex of KEAP1 and the mitochondrial outer membrane serine/threonine protein phosphatase, PGAM5 [29, 30]. This NFE2L2-KEAP1-PGAM5 complex has been reported to play a role in mitochondrial retrograde trafficking. A decrease in NFE2L2 or PGAM5 expression results in decreased mitochondrial motility, which is particularly important for the transport of mitochondria along the neuronal axon [29]. Furthermore, PGAM5 is also a binding protein of the antiapoptotic protein, BCL-XL [31]. A decrease in PGAM5 may lead to KEAP1-mediated BCL-XL degradation, which thereby promotes apoptosis [32]. In particular, considering the reduction of NFE2L2 or PGAM5 in aging and human degenerative disease states [32–35], this NFE2L2-KEAP1-PGAM5 ternary interaction may be an important mechanism in the development of human diseases.

## 4. Mitochondrial Homeostasis and Dynamics

**Figure 1:**
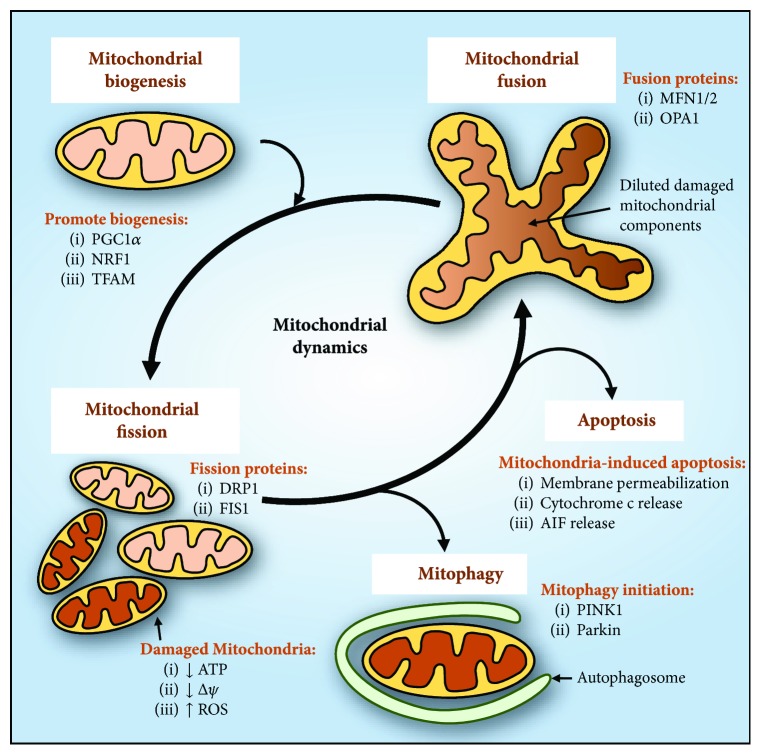
Mitochondrial homeostasis is dynamically maintained by the processes of mitochondrial biogenesis, mitochondrial fusion/fission, mitophagy, and apoptosis. The upregulation of peroxisome proliferator-activated receptor *γ* coactivator-1*α* (PGC1*α*), nuclear respiratory factor 1 (NRF1), and mitochondrial transcription factor A (TFAM) promotes mitochondrial biogenesis. In mammals, mitochondrial fusion is facilitated by mitofusin (MFN) 1 and 2 and OPA1 for the fusion of the outer and inner mitochondrial membranes, respectively. Mitochondrial fission involves dynamin-related protein 1 (DRP1) that interacts with fission protein 1 (FIS1), which compartmentalizes damaged mitochondrial components into daughter mitochondria for elimination *via* mitophagy. Decreased ATP levels and membrane potential (*∆ψ*) and increased ROS generation are features of damaged mitochondria. These dysfunctional mitochondria are detected by phosphatase and tensin homologue deleted on chromosome 10- (PTEN-) induced putative kinase 1 (PINK1) and recruits Parkin, which initiates mitophagy and the subsequent formation of the autophagosome to degrade targeted mitochondria. Damaged mitochondria can also induce apoptosis through the permeabilization of the mitochondrial membrane, leading to the release of cytochrome *c* that can activate caspase-mediated apoptosis, as well as the release of proapoptotic proteins such as apoptosis-inducing factor (AIF).

The maintenance of mitochondrial homeostasis is critical for proper functioning of the cell. Hence, mitochondria have a network of dynamic processes that tightly regulate its homeostasis and life cycle, namely, mitochondrial fusion and fission, mitophagy, and mitochondrial biogenesis (Figure 1) [36–38]. Mitochondrial fusion and fission mediate mitochondrial quality control through regulation of its turnover *via* mitochondrial biogenesis and elimination [37, 39].

### 4.1. Mitochondrial Fusion

Mitochondrial fusion is a dynamic process in which two or more mitochondria fuse together in an attempt to reduce mitochondrial stress that could be induced by senescent or damaged proteins and ROS (Figure 1) [39, 40]. This process enables damaged mitochondria to repair their function and prevent the accumulation of mtDNA mutations [39]. Mitochondrial fusion requires a spatially coordinated fusion of the outer and inner mitochondrial membranes that are different in electrophysiological properties, structure, and composition [40]. Notably in mammals, fusion of the outer and inner mitochondrial membranes is facilitated by members of the membrane-anchored dynamin family, mitofusin (MFN) 1 and 2, and the single dynamin family member, OPA1, respectively [36, 41, 42].

### 4.2. Mitochondrial Fission

When mitochondrial fusion is unable to restore mitochondrial homeostasis in disease conditions, the dynamic nature of the mitochondrial network shifts towards mitochondrial fission which leads to the removal of damaged mitochondria (Figure 1) [39]. Mitochondrial fission compartmentalizes damaged mitochondrial components into daughter organelles that are to be removed and targeted for elimination [39]. In mammals, mitochondrial fission involves the cytoplasmic protein, dynamin-related protein 1 (DRP1), which forms a ring structure to encircle and constrict at a site on the outer mitochondrial membrane upon its interaction with fission protein 1 (FIS1) [39, 43]. As a result, mitochondrial fission generates smaller and spherical mitochondria, as opposed to the tubular morphologies observed from mitochondrial fusion [39].

### 4.3. Mitophagy

**Figure 2:**
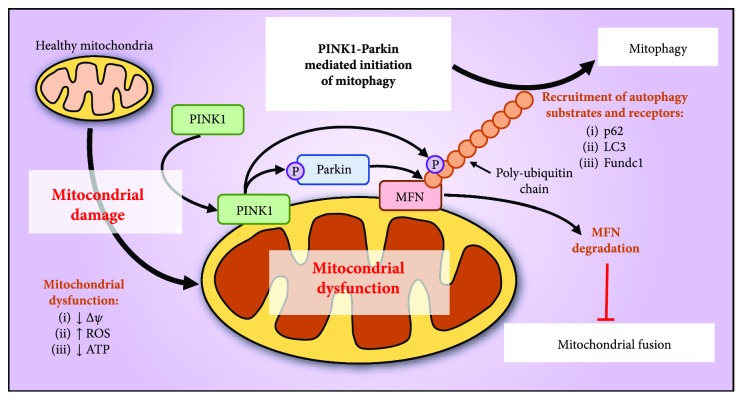
Phosphatase and tensin homologue deleted on chromosome 10- (PTEN-) induced putative kinase 1- (PINK1-) Parkin mediated initiation of mitophagy and inhibition of mitochondrial fusion. PINK1 recognizes damaged mitochondria that exhibit mitochondrial dysfunction. As a result, PINK1 accumulates on the outer mitochondrial membrane, which recruits and activates Parkin *via* its phosphorylation at Ser65 on the *N*-terminal ubiquitin-like domain and phosphorylates ubiquitin. Phosphorylated Parkin then recruits and forms ubiquitin chains on mitofusin (MFN) located on the outer mitochondrial membrane, leading to its proteasomal degradation and inhibition of mitochondrial fusion. As such, the ubiquitination of MFN promotes mitophagy through the recruitment of autophagy substrates and receptors such as p62, LC3, and Fundc1 that facilitates the elimination of the targeted mitochondria.

In response to mitochondrial stress, mitochondrial fusion and fission also play an important role in the elimination of irreversibly damaged mitochondria through an autophagic process known as mitophagy [39, 44]. The mechanism of mitophagy has been attributed to a number of key molecules, particularly phosphatase and tensin homologue deleted on chromosome 10- (PTEN-) induced putative kinase 1 (PINK1) and Parkin which were identified in models of Parkinson's disease (PD) [45–47]. PINK1 is a serine/threonine kinase that specifically targets mitochondria while Parkin is an E3 ubiquitin ligase, with mutations in either genes resulting in the early-onset autosomal recessive form of PD [46, 48]. The initiation of mitophagy involves the targeting of damaged mitochondria by PINK1 that recruits and activates Parkin *via* its phosphorylation at Ser65 on the *N*-terminal ubiquitin-like domain (Figure 2) [49, 50]. PINK1 also phosphorylates ubiquitin at Ser65 leading to structurally distinctive properties, which allows for interactions with ubiquitin-binding proteins specific for mitophagy [49–51]. The phosphorylation of Parkin and ubiquitin by PINK1 leads to the recruitment and subsequent formation of ubiquitin chains on outer mitochondrial membrane proteins, such as MFN1 and/or MFN2 (Figure 2) [49]. The ubiquitination of MFN results in the inhibition of mitochondrial fusion and the recruitment of autophagy receptors to promote mitophagy (Figure 2) [45, 49, 52]. Therefore, the interaction between PINK1 and Parkin is critical for the initiation and regulation of mitophagy.

However, PINK1-independent mechanisms may exist as demonstrated by a recent study where PINK1 deficiency does not inhibit basal mitophagy in multiple high energy–demanding tissues, including neural tissue and the heart [53]. Over the past decade, a number of mitochondrial-localized mitophagic markers that interact with the autophagosomal protein, microtubule-associated protein 1A/1B-light chain 3 (LC3), have also been identified [49, 54–56]. These include FUNDC1, BNIP3, NIX, optineurin, and NDP52, which also potentiate mitophagy through their LC3-interacting regions (LIR) in both a PINK1-dependent and independent manner [49, 54–56]. A recent addition to this list of mitophagic markers is AMBRA1 [57]. AMBRA1 mediates the mitochondrial localization of the ubiquitin ligase HUWE1 and potentiates MFN2 ubiquitination and degradation but also the recruitment of autophagosome *via* the AMBRA1 LIR motif [57].

### 4.4. Mitochondrial Biogenesis

In addition to the removal and processing of mitochondrial stress, there is a need for the restoration of mitochondrial deficits by producing new mitochondria through mitochondrial biogenesis. This results in the replication of mtDNA and the synthesis and assembly of mitochondrial components. The transcription coactivator, PGC1*α*, regulates mitochondrial biogenesis by activating a group of transcription factors, such as NRF1, and the mitochondrial transcription factor A (TFAM) [58]. These two transcription factors mediate the transcription of nuclear DNA and mtDNA, respectively [58].

Together, the dynamic processes of mitochondrial fusion and fission, mitophagy, and biogenesis act to restore normal mitochondrial function and morphology in the presence of mitochondrial stress and damage, thus maintaining mitochondrial homeostasis.

## 5. Mitochondria and Apoptosis

Apoptosis is an active mechanism of programmed cell death in response to stress-inducing or regulatory signals. This process is tightly regulated to facilitate the growth, development, and replication or replacement of cells to maintain a normal cellular life cycle. Impairment of mitochondrial function and structure destabilizes the cell and initiates a signaling cascade for apoptosis [59]. There are a number of mechanisms by which mitochondria induce and mediate the process of programmed cell death in mammals. This often involves the permeabilization of the mitochondrial membrane with the release of cytochrome *c* and proapoptotic proteins that causes a cascade of apoptotic signaling to execute apoptosis. Mitochondrial mechanisms for apoptosis can be caspase-dependent or -independent (for more detail, see [8, 60–62]).

**Figure 3:**
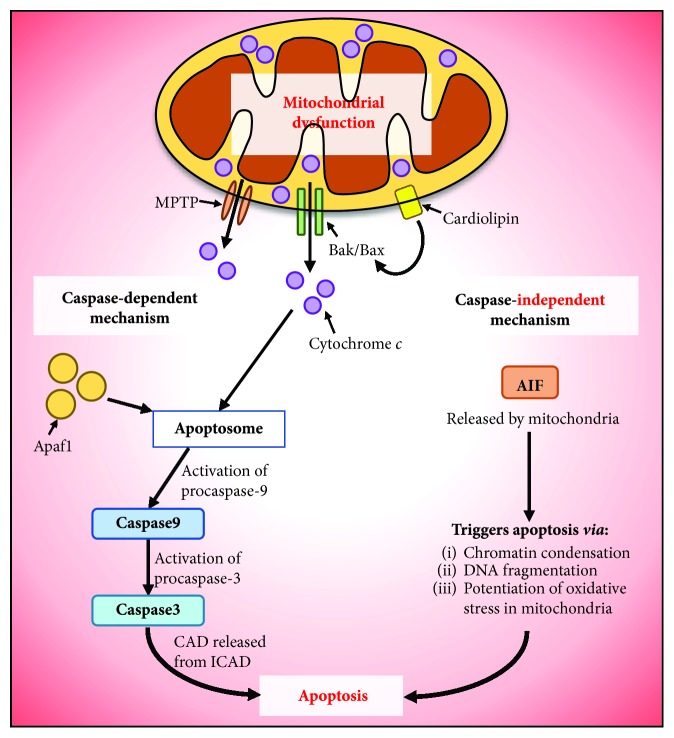
Mitochondrial caspase-dependent and caspase-independent mechanisms of apoptosis. Mitochondrial dysfunction leads to the permeabilization of its membranes, which is the first step towards apoptosis. Membrane permeabilization of the outer mitochondrial membrane is driven by the mitochondrial permeability transition pore (MPTP), members of the BCL2 protein family (i.e., BAK/BAX), and mitochondrial lipids such as cardiolipin. More specifically, cardiolipin is associated with BAX recruitment to the outer mitochondrial membrane that triggers membrane permeabilization. For the caspase-dependent mechanism of apoptosis, mitochondrial cytochrome *c* is released to trigger the formation of the apoptosome complex by binding to, and activating, the apoptotic protease activating factor 1 (Apaf1). This in turn, activates caspase-9 and -3, which leads to the release of CAD from its inhibitor, ICAD, resulting in apoptosis induction. The caspase-independent mechanism of apoptosis involves the mitochondrial release of proapoptotic proteins such as apoptosis-inducing factor (AIF) into the cytosol, whereby it can either directly interact with DNA or potentiate mitochondrial oxidative stress through its release to induce apoptosis.

Cytochrome *c* is an essential component of the respiratory chain that facilitates the transfer of electrons from complex III to complex IV [62]. Mitochondrial dysfunction, mitochondrial membrane permeabilization, and oxidative stress can disrupt the electron transport chain and affect cytochrome *c* function [8]. In response, mitochondria release cytochrome *c* to the cytosol to trigger downstream activation of caspases and the formation of a caspase-activated complex, the apoptosome, which leads to apoptosis with the degradation of cellular components (Figure 3) [60]. The release of cytochrome *c* is mediated by protein members of the B-cell lymphoma 2 (BCL2) family, such as BAK and BAX, the mitochondrial permeability transition pore (MPTP), and mitochondrial lipids to execute apoptosis (Figure 3) [8]. Additionally, cytochrome *c* activates caspase-3 and -9 in the cytosol *via* forming the apoptosome complex by binding to and activating the apoptotic protease factor 1 (Apaf1) [63]. It is well established that the activation of caspase-3, in turn, liberates the caspase-activated deoxyribonuclease (CAD) from its inhibitor, ICAD, which results in apoptotic features of DNA fragmentation and chromatin condensation (Figure 3) [8, 64, 65].

Oxidized lipids also play an important role in the induction of apoptosis [66, 67]. Cardiolipin is the mitochondria-specific lipid whose oxidation results in mitochondrial membrane permeability and the recruitment of the proapoptotic protein, BAX (Figure 3) [66, 68, 69]. Cytochrome *c* is normally associated with cardiolipin in the inner mitochondrial membrane [70, 71]. The oxidation of cardiolipin results in both mitochondrial membrane permeabilization and cytochrome *c* dissociation and release [70–72].

Alternatively, following mitochondrial dysfunction, oxidative stress, or a decrease in ATP levels, a caspase-independent mechanism of mitochondrial-associated apoptosis may also be induced [60]. This involves permeabilization of the outer and inner mitochondrial membranes, whereby the mitochondria releases proapoptotic proteins, such as apoptosis-inducing factor (AIF), into the cytosol to regulate apoptosis (Figure 3) [60]. The translocation of AIF from the mitochondria to the cytosol occurs in a BCL2-controlled manner in which cytosolic AIF can travel further into the nucleus where it causes DNA fragmentation and chromatin condensation (Figure 3) [73, 74]. Furthermore, the mitochondrial release of AIF can also increase oxidative stress due its potential role in maintaining ROS levels generated by the respiratory chain (Figure 3) [73].

Previous studies on diabetic neuronal injury have also shown a mitochondrial profile of decreased mitochondrial membrane potential and BCL2 expression, accompanied by ROS generation and increased expression of proapoptotic proteins [75]. Similar mitochondrial alterations that mediate apoptosis are found in cardiac aging and pulmonary hypertension (for reviews: [76, 77]). In many of these diseases, mitochondrial oxidative stress appears to be a key feature of mitochondrial dysfunction that drives apoptosis in disease progression.

## 6. Iron Homeostasis and Mitochondrial Dysfunction

Iron is the most abundant transition metal in mammalian cells and is essential for myriad biological processes, including oxygen transport, cellular respiration, and DNA synthesis/repair [78]. The mitochondrion is a major site of iron metabolism, particularly the synthesis of heme (Fe-protoporphyrin IX) and ISCs that are essential cofactors required by the electron transport chain [1]. In terms of the delivery of iron into the mitochondrion, the only known iron transport protein that imports iron across the inner mitochondrial membrane is mitoferrin (MFRN) [79–81].

Two MFRNs exist: MFRN1 is erythroid-specific, while MFRN2 is ubiquitously expressed with low expression in erythroid cells [79]. Other potential mechanisms of iron delivery to the mitochondria have recently come to light. These involve glutaredoxin 3 [82, 83], or endocytic mechanisms (i.e., the “kiss and run” hypothesis) of targeted mitochondrial iron delivery *via* direct endosomal-mitochondrial contact that results in the metal ion bypassing the cytosol [84, 85]. Other mitochondrial proteins may also be involved in mitochondrial iron import. An example is the inner mitochondrial membrane ATP-binding cassette (ABC) transporter ABCB10, which physically interacts with MFRN1 to stabilize MFRN1 and increase mitochondrial iron import into the erythron [86].

## 7. Mitochondrial Dysfunction in Neurodegenerative Diseases

Neurons have a high metabolic load that is demonstrated by the fact that although the brain only accounts for 2% of human body mass, it consumes 20% of the body's resting ATP production [87]. Studies over the past decade have demonstrated that neurodegenerative disorders manifest common pathological events associated with mitochondria. These include mitochondrial dysfunction [3], oxidative stress, autophagic dysfunction, and apoptosis [88]. In fact, defects and mutations within the genome are often pathological causes of many degenerative diseases that alter mitochondrial function.

### 7.1. Alzheimer's Disease (AD)

**Figure 4:**
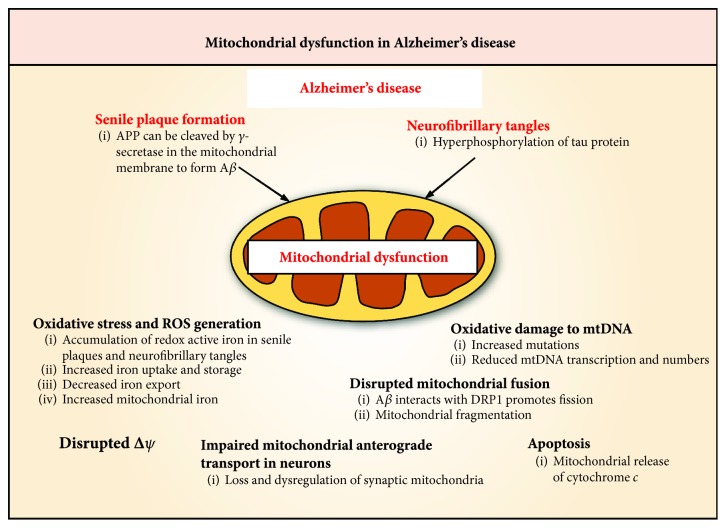
Mitochondrial dysfunction in the pathogenesis of Alzheimer's disease (AD). Hallmarks of AD include the formation of senile plaques composed of *β*-amyloid (A*β*) and neurofibrillary tangles caused by tau hyperphosphorylation. Amyloid precursor protein (APP) has been reported to translocate and accumulate in mitochondrial membranes and could be cleaved by *γ*-secretase to form A*β*, leading to mitochondrial dysfunction. The accumulation of redox active iron in senile plaques and neurofibrillary tangles, as well as the overall increased iron levels in mitochondria, leads to ROS generation and oxidative stress. The mitochondrial membrane potential (*∆ψ*) is also disrupted in AD. Mitochondrial DNA (mtDNA) suffers oxidative damage in which there are increased mutations to mtDNA with reduced transcription and mtDNA number. Mitochondria in AD also have disrupted mitochondrial fusion whereby the interaction between A*β* and DRP1 promotes mitochondrial fission and subsequent mitochondrial fragmentation. In neurons, there is the loss and dysregulation of synaptic mitochondria, which leads to the impairment of mitochondrial anterograde transport. Finally, mitochondrial dysfunction in AD can lead to apoptosis through cytochrome *c* release.

Alzheimer's disease (AD) is the most common neurodegenerative disease, with an estimated 46.8 million AD patients worldwide [89]. AD is clinically characterized by progressive cognitive decline associated with senile plaques composed of *β*-amyloid (A*β*) peptide and neurofibrillary tangles composed of hyperphosphorylated tau [90]. In fact, mitochondrial dysfunction is a characteristic of A*β*-induced neurotoxicity in AD [90]. It has been reported that the amyloid precursor protein (APP) could translocate and accumulate in the mitochondrial membrane [90], where it may be cleaved by *γ*-secretase forming the toxic A*β* peptide [91, 92]. Subsequently, the A*β* peptide interacts with a number of mitochondrial proteins, which disrupts mitochondrial membrane potential and promotes apoptosis *via* cytochrome *c* release (Figure 4) [93, 94].

The pathogenesis of AD likely involves oxidative damage to mtDNA [95]. When AD patient mtDNA is inserted into mtDNA-deficient cells, the resulting cybrids showed respiratory enzyme defects and elevated ROS production and free radical scavenging enzyme activities that were seen in AD patient brains [95]. Regulatory regions in mtDNA from AD brains showed increased mutations relative to controls [96]. These mutations lead to an average 50% reduction in mtDNA transcription and mtDNA copy number, potentiating mitochondrial dysfunction (Figure 4) [96]. The ensuing ROS generation due to mitochondrial dysfunction in AD is well documented and leads to activation of the NFE2L2 pathway [97, 98]. Pharmacological targeting of NFE2L2 was found to elicit neuroprotection in A*β*-induced hippocampal neuron injury and appeared to involve the activation of the NFE2L2 downstream target, HO-1 [97, 98]. Additionally, pharmacological targeting of KEAP1 and GSK3*β* that regulate NFE2L2 activity resulted in neuroprotection in a mouse model of tauopathy [99].

It has been reported that A*β* disrupts mitochondrial fusion, resulting in mitochondrial fragmentation [100, 101]. In AD brains, increased A*β* production and its interaction with DRP1 are crucial factors causing mitochondrial fission and neuronal damage (Figure 4) [102]. Conversely, reduced DRP1 expression or inhibition of DRP1 with a mitochondrial division inhibitor (mdivi1) restored pathologic A*β*- or tau-mediated mitochondrial fragmentation, mitochondrial dysfunction, and synaptic depression in neurons [103]. Furthermore, inhibition of DRP1 decreased *β*-secretase 1 (BACE1) expression and A*β* deposition in the brain of AD mice, leading to a concomitant increase in cognitive function [104, 105].

The loss of synapses in AD brains correlates strongly with a cognitive decline [106, 107]. A recent study demonstrated that the loss and dysregulation of synaptic mitochondria may be an important pathogenic factor in AD progression [108]. The synapse is a region of high energy demand and requires constant trafficking of mitochondria to this region [109]. As tau is involved in stabilizing microtubules required for anterograde transport of mitochondria (Figure 4) [109], tau hyperphosphorylation destabilizes microtubules and impairs mitochondrial anterograde transport [110, 111]. Moreover, oligomeric A*β* has also been shown to impair mitochondrial motility in hippocampal neurons without destabilizing microtubules [112–114]. This latter effect may potentially involve NFE2L2 and KEAP1's role in mitochondrial motility [29], since depletion of NFE2L2 inhibits mitochondrial motility [29] and NFE2L2 induction elicits neuroprotection in AD models [97–99, 115].

In AD, excessive ROS generation caused by A*β* exacerbates mitochondrial dysfunction and redox imbalance within neurons, which leads to neuronal damage [116, 117]. As a result, mitochondria suffer membrane depolarization, calcium overload, and cytochrome *c* release, which collectively induces apoptosis [117, 118]. In a different study examining AD pathology, overexpression of APP induced mitochondrial oxidative stress that triggers mitochondrial membrane permeabilization and cytochrome *c* release [119]. This suggests an important apoptotic role of mitochondria in the pathophysiology of AD.

The accumulation of redox-active iron in senile plaques and neurofibrillary tangles is another facet of AD pathology (Figure 4) [120–122]. Studies of AD models demonstrated increased iron uptake and storage with reduced iron export [123–125]. Indeed, APP mRNA has an atypical, but functional ferritin-like iron responsive element, and thus, an increase in intracellular iron level enhances *APP* mRNA translation *via* the iron regulatory element/iron regulatory protein system [126]. Recent studies demonstrated that the knockdown of *MFRN1* in a *C. elegans* model of AD reduced mitochondrial iron content and mitochondrial ROS and resulted in increased lifespan [127]. This is supported by studies demonstrating that overexpression of mitochondrial ferritin (FtMt) attenuates A*β*-induced neuronal apoptosis [128], while A*β*-induced cognitive decline and neuronal apoptosis were exacerbated in FtMt KO mice relative to WT mice [129]. These findings suggest that increased iron uptake in AD neurons leads to increased mitochondrial iron loading that may exacerbate the pathogenesis of the disease.

### 7.2. Parkinson's Disease (PD)

Parkinson's disease (PD) is the second most common neurodegenerative disease after AD and affects 1% of the population above 60 years of age [130]. PD is clinically characterized by motor dysfunction, including muscle rigidity, bradykinesia, and resting tremor, as well as nonmotor symptoms, such as dementia [131]. The major pathological feature of PD is the loss of dopaminergic neurons and the accumulation of *α*-synuclein-containing Lewy bodies in the substantia nigra [131]. In the majority of PD cases, the cause is unknown, although a number of familial PD cases have been identified due to mutations in genes that are involved in mitochondrial homeostasis [131]. A prominent feature of PD pathology is the inhibition of the activity of mitochondrial complexes I and IV in dopaminergic neurons of the substantia nigra [132–135]. This could be associated with a dysregulation of mitochondrial genome maintenance [136] or a number of PD-associated molecules discussed below.

**Figure 5:**
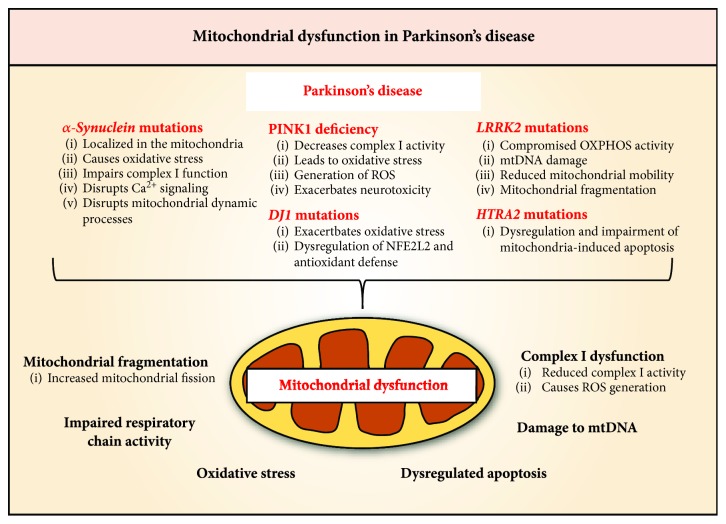
The different causes of mitochondrial dysfunction in the pathology of familial Parkinson's diseases (PD). Some familial cases of PD include mutations in *α-synuclein*, PINK1-deficiency, and mutations in *DJ1* (*PARK7*), *leucine-rich repeat kinase 2* (*LRRK2*), and *high-temperature requirement protein A2* (*HTRA2*). Mutations in *α-synuclein* result in the protein becoming localized in the mitochondria, causing mitochondrial dysfunction *via* oxidative stress, impaired Ca^2+^ signaling, complex I dysfunction, and mitochondrial fragmentation. PINK1 deficiency and mutations in *LRRK2* also lead to impaired respiratory chain activity. Furthermore, *LRRK2* mutations can reduce mitochondrial mobility and cause mtDNA damage. Mutations in *DJ1* affect its role to regulate NFE2L2 degradation, resulting in the potential exacerbation of oxidative stress. Furthermore, *HTRA2* mutations lead to the dysregulation and impairment of mitochondria-induced apoptosis.

The *α*-synuclein protein is critical for the recycling of vesicles at the presynaptic membrane [137]. In the dopaminergic neurons, *α*-synuclein plays a critical role for the synthesis, regulation, storage, and release of dopamine [138]. Mutations in *α-synuclein* are associated with highly penetrant, autosomal dominant, familial PD [139]. In dopaminergic neurons, overexpression of WT or mutant *α*-synuclein reduces dopamine release, potentiates the formation of toxic *α*-synuclein oligomers, and results in dopamine-dependent neurotoxicity [138, 140, 141]. Aggregation of *α*-synuclein into Lewy bodies is a prominent pathological feature in PD and other neurodegenerative disorders that are collectively known as *α*-synucleinopathies [142]. The *α*-synuclein protein can be imported into mitochondria and associates with the inner mitochondrial membrane of dopaminergic neurons [143–145]. Overexpression of *α*-synuclein exacerbates mitochondrial dysfunction, oxidative stress, and neuropathology caused by complex I inhibition (Figure 5) [146], while *α*-synuclein deficiency attenuates these effects [147, 148]. Therefore, it is speculated that the interaction between aggregated *α*-synuclein with mitochondrial respiratory complex I leads to the impairment of this complex [144].

In addition, oligomeric *α*-synuclein, or the A53T mutant form, has been demonstrated to interact with outer mitochondrial membrane proteins, including translocase of the outer membrane 20 (TOM20) and voltage-dependent anion-selective channel 1 (VDAC1), to block the import of mitochondrial proteins/metabolites [149, 150] or inhibit mitochondria-ER interactions to disrupt Ca^2+^ signaling [151, 152]. Moreover, while monomeric *α*-synuclein has also been shown to interact with ATP synthase to improve ATP production [153], the aggregated *α*-synuclein induces the opening of mitochondrial permeability transition pore (PTP), resulting in mitochondrial swelling and cell death [154]. Furthermore, aggregated or mutant *α*-synuclein also impairs the mitochondrial network fission/fusion processes [155] and subsequent mitophagy (Figure 5) [156], possibly *via* regulation of the actin cytoskeleton [157–159]. A recent study by Grassi and colleagues identified a novel and highly neurotoxic form of *α*-synuclein that results from incomplete autophagic degradation that associates with mitochondria and induces mitochondrial toxicity and fragmentation [160].

Mutations within *PINK1* cause autosomal recessive juvenile PD [47]. PINK1 selectively accumulates in dysfunctional mitochondria [161]. Overexpression of PINK1 in neurons prevents apoptosis by decreasing cytochrome *c* release and the activation of caspases [162]. In models of PD, PINK1 overexpression suppresses *α*-synuclein-induced toxicity, potentially *via* the induction of autophagic *α*-synuclein removal [155, 163, 164], whereas PINK1 deficiency exacerbates the neurotoxicity of aggregated *α*-synuclein (Figure 5) [165, 166]. PINK1 expression is also increased following *α*-synuclein overexpression, suggesting a protective role of PINK1 in PD [164].

Mutation of the *DJ1* (*PARK7*) gene encoding a protein deglycase is associated with autosomal recessive juvenile PD [167]. DJ1 is suggested to regulate oxidant defenses [168, 169] and participate in the formation of mitochondrial complex I [170]. DJ1 has also been shown to interact with monomeric or oligomeric *α*-synuclein to inhibit oligomer formation and prevent toxicity [171, 172]. DJ1 may also interact with PINK1/Parkin [173, 174], but this was disputed in a later study [175]. However, the consensus is that DJ1 mutation or deletion results in dysfunctional mitophagy that may act in parallel to the PINK1/Parkin pathway [175–177]. Oxidative stress results in the acidification of a critical cysteine residue (C106) of DJ1, leading to its localization to the mitochondrion where it exerts a neuroprotective effect [178]. In fact, DJ1 was found to stabilize NFE2L2 by preventing its association with KEAP1 and subsequent NFE2L2 degradation [179]. Therefore, *DJ1* mutations could lead to the dysregulation of NFE2L2 and the antioxidative response in PD (Figure 5).

Activators of NFE2L2 have been found to be neuroprotective in PD models caused by 1-methyl-4-phenyl-1,2,3,6-tetrahydropyridine- (MPTP-) induced complex I inhibition [180–182]. In a multicenter study, a *NFE2L2* haplotype associated with high transcriptional activity was found to significantly decrease disease risk and delay the onset of idiopathic PD [183]. Moreover, while mitochondrial membrane potential is greatly reduced in dopaminergic neurons from PINK1-KO mice, treatment with NFE2L2 activators is able to completely rescue this defect, as well as being protective against dopamine-induced neuronal death [184]. This finding suggests that NFE2L2 activation may be a viable therapeutic avenue in PINK1-associated PD.

Mutations in *leucine-rich repeat kinase 2* (*LRRK2*) are the most common cause of autosomal dominant familial PD as well as some cases of sporadic PD [185, 186]. PD patient-derived cells carrying a *LRRK2* mutation resulted in compromised OXPHOS activity, mtDNA damage, and reduced mitochondrial motility with increased mitochondrial fragmentation (Figure 5) [187–189]. These pathologic effects are dependent on LRRK2 kinase activity and can be reversed by LRRK2 kinase inhibitors [190, 191]. The LRRK2 is a serine-threonine kinase that has been demonstrated to associate with the outer mitochondrial membrane [192], potentially through its interaction with a key regulator of mitochondrial fission, DRP1 [193, 194]. Indeed, PD-associated mutations in *LRRK2* kinase domain increases its catalytic activity [192, 195] which results in increased DRP1 Ser616 phosphorylation and activation of mitochondrial fission [188, 189]. These results suggest that the pathogenesis of both familial and sporadic PD associated with *LRRK2* mutations may involve a direct perturbation of mitochondrial fission.

High-temperature requirement protein A2 (HTRA2) is a serine protease in the mitochondrial intermembrane space [196]. Disruption to *HTRA2* has been associated with increased risk of sporadic PD [197, 198]. HTRA2 is important for mitochondrial quality control and is responsible for the degradation of denatured proteins within the mitochondria [199]. Following apoptotic stimuli, HTRA2 is released from the intermembrane space and binds to the inhibitor of apoptosis proteins (IAP) [196]. Subsequently, HTRA2 induces caspase activity and caspase-independent death through its protease activity (Figure 5) [196]. Recent studies have demonstrated that HTRA2 exerts its neuroprotective effect by targeting *DJ1* mutations, thereby linking the two genetic factors of PD [200].

Previous studies in models of PD have also demonstrated the involvement of mitochondria in the apoptosis of dopaminergic neurons [201, 202]. In PD, studies have shown that depolarization of mitochondria results in reduced mitochondrial membrane potential and is associated with the early stages of apoptosis [59].

### 7.3. Amyotrophic Lateral Sclerosis (ALS)

Amyotrophic lateral sclerosis (ALS) is a lethal neurodegenerative disorder characterized by progressive degeneration of upper and lower motor neurons [203]. The prevalence of ALS is approximately 4-6 in 100,000 individuals [204]. Approximately 10% of ALS are familial cases, of which about 20% are due to autosomal dominant mutations in *Cu/Zn-superoxide dismutase* (*SOD1*), which is a major antioxidant enzyme [205]. Indeed, according to the review by Smith et al., there are at least 11 pathogenic variants of proteins and their respective ALS-associated genes that have the potential to affect mitochondrial function, hence demonstrating the significance of mitochondrial dysfunction in the pathophysiology of ALS [204].

**Figure 6:**
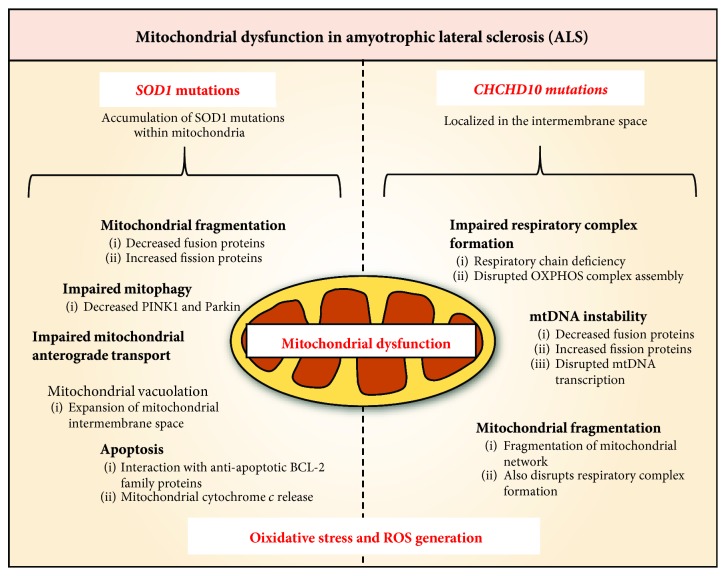
Mutations in *superoxide dismutase* (*SOD1*) and *coiled-coil-helix-coiled-coil helix domain 10* (*CHCHD10*) cause mitochondrial dysfunction in familial cases of amyotrophic lateral sclerosis (ALS). *SOD1* mutations cause a number of mitochondrial defects including mitochondrial fragmentation, impaired mitophagy, and impaired mitochondrial anterograde transport of mitochondria, mitochondrial vacuolation, and apoptosis. CHCHD10 is localized in the mitochondrial intermembrane space, and mutation of the gene encoding this protein leads to a defect in the formation of the respiratory complex, mtDNA instability, and fragmentation of the mitochondrial network. Overall, oxidative stress is a common feature in ALS pathology. This is potentially due to the disruption of the respiratory chain caused by mitochondrial dysfunction.

An accumulation of swollen and vacuolated mitochondria with abnormal cristae was one of the first pathological features observed in ALS patient motor neurons [206]. This is recapitulated in animal and cellular models of ALS, where a proliferation of swollen and fragmented mitochondria is frequently observed [207–210]. This process may involve reduced mitochondrial fusion proteins (e.g., MFN1/2, OPA1), increased fission proteins (e.g., DRP1, FIS1), or impaired mitophagy (decreased PINK1, PARKIN) [211–214]. The ALS-associated *SOD1* mutation results in an accumulation of misfolded SOD1 in axonal mitochondria of motor neurons [215] and an impaired anterograde axonal transport of mitochondria [207, 215] that is mirrored in other models of familial ALS (Figure 6) [207, 216].

The interaction of ALS-associated mutant protein with the mitochondria is a major cause of mitochondrial damage. In fact, aggregation of mutant SOD1 within mitochondria causes mitochondrial vacuolation through expansion of the intermembrane space (Figure 6) [217, 218]. This may be caused by an interference of the SOD1 aggregates with VDAC1 that interrupts the exchange of vital substrates such as ADP across the outer mitochondrial membrane [219, 220]. In addition, ALS mutant SOD1 has been found to interact with antiapoptotic BCL2 specifically in the spinal cord (Figure 6) [221, 222]. This causes a proapoptotic conformational change in BCL2 that exposes its toxic BH3 domain and compromises mitochondrial membrane integrity and results in cytochrome *c* release [222]. The mutant SOD1-BCL2 complex prevents the interaction between BCL2 and VDAC1 and thus reduces the permeability of the outer mitochondrial membrane [223].

Moreover, a number of mutations in the genes encoding DNA/RNA-binding proteins have recently been associated with both familial and sporadic ALS [207, 209, 210, 216, 224]. These include TDP43, TARDBP, C9ORF72, and FUS proteins [207, 209, 210, 216, 224]. The interaction of these ALS mutant proteins with mtDNA transcripts disrupts their transcription and impairs the formation of the respiratory complex [210, 224, 225]. On the other hand, a newly identified mutation in the gene encoding the mitochondrial protein, coiled-coil-helix-coiled-coil helix domain 10 (CHCHD10), causes ALS-like symptoms in humans and is characterized by mtDNA instability, respiratory chain deficiency, and mitochondrial network fragmentation (Figure 6) [226]. CHCHD10 is localized in the mitochondrial intermembrane space, and it is enriched at cristae junctions within the mitochondrial contact site and cristae organizing system (MICOS) complex [226, 227]. Mutant *CHCHD10* leads to fragmentation of the mitochondrial network, disassembly of the MICOS complex that disrupts the assembly of OXPHOS complexes, and decreased nucleoid number and nucleoid disorganization that potentiates mtDNA instability (Figure 6) [226, 227]. Disruptions to these crucial mitochondrial components ultimately impair mitochondrial function and potentiate the ROS generation reported in ALS patients (Figure 6) [228].

### 7.4. Huntington's Disease (HD)

**Figure 7:**
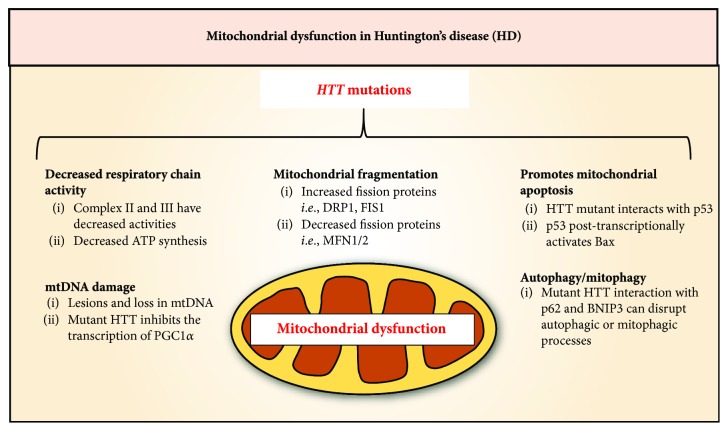
Mitochondrial dysfunction in the pathology of Huntington's disease (HD). A CAG trinucleotide repeat expansion in the *huntingtin (HTT)* gene causes an array of mitochondrial dysfunctions in HD. Mutations in *HTT* are associated with a decrease in ATP synthesis and complex II and II activities. Mutant HTT also promotes mitochondrial apoptosis *via* its interaction with p53, leading to its enhanced expression and transcriptional activity that activates proapoptotic BAX. Damage and loss of mtDNA is another feature of HD that is attributed to the inhibition of PGC1*α* transcription by mutant HTT. Moreover, mutations in *HTT* contribute to mitochondrial fragmentation through the upregulation of mitochondrial fission proteins such as DRP1 and FIS1 and the downregulation of the fusion proteins, MFN1/2. Lastly, mutant HTT proteins can disrupt autophagy or mitophagy through its interaction with the autophagic adaptor, p62, and the mitophagic protein, BNIP3, respectively.

HD is an autosomal dominant progressive neurodegenerative disorder clinically characterized by chorea, dystonia, incoordination, and cognitive decline [229]. The prevalence of HD is estimated to be 10.6-13.7 individuals per 100,000 people in Western populations [230]. HD is caused by a CAG trinucleotide repeat expansion in the *huntingtin* (*HTT*) gene, resulting in polyglutamine repeats in the HTT protein [229]. The activity of respiratory complexes II and III is decreased in HD (Figure 7) [231]. In a mutant HD mouse model, mitochondrial respiration and ATP synthesis are significantly decreased (Figure 7) [232]. In mammals, chronic administration of complex II inhibitors replicates many clinical features of HD [233–235]. Conversely, the protein, rather than mRNA, expression of two important constituents of mitochondrial complex II, the 30 kDa iron-sulfur (Ip) subunit and the 70 kDa FAD (Fp) subunit, is preferentially decreased in the striatum of HD patients [236]. Overexpression of either complex II subunits restores complex II activity and attenuates mitochondrial dysfunction and death in mutant HTT neuronal cells [236].

HTT has been found to be associated directly with the outer mitochondrial membrane [237, 238]. Mitochondria from HD patient lymphoblasts or from a HD mouse model have a lower membrane potential and become depolarized at lower Ca^2+^ loads than relevant controls [237, 238]. Mutant HTT may also affect mitochondrial function through its interaction with transcription factors, such as p53, CREB-binding protein and specificity protein 1 [239]. Of note, p53 activates mitochondrial apoptosis through transcriptional induction of p53 upregulated modulator of apoptosis (PUMA) or the posttranscriptional activation of BAX (Figure 7) [240, 241]. In neuronal cultures, mutant HTT binds p53 and enhances nuclear p53 expression and its transcriptional activity [242]. Conversely, p53 inhibition in mutant HTT fly and mouse models attenuates HTT-mediated neurodegeneration [242].

The pathogenesis of HD also involves increased mtDNA lesions and mtDNA depletion [243]. This pathological alteration could be attributed to decreased *PGC1α* mRNA observed in early-stage HD patients [244, 245]. Mutant HTT directly inhibits transcription of PGC1*α* by associating with the promoter region and interfering with the activation functions of the transcription factors CREB and TAF4 (Figure 7) [244]. Moreover, overexpression of PGC1*α* partially attenuated mutant HTT-induced neurotoxicity [244]. In contrast, PGC1*α* KO mice demonstrate impaired mitochondrial function and possess HD features such as a hyperkinetic movement disorder and striatal neuron degeneration [246].

Mutant HTT protein also potentiates mitochondrial fragmentation [247]. This occurs through the induction and activation of mitochondrial fission regulators DRP1 and FIS1, while reducing the expression of fusion proteins, such as MFN1/2 (Figure 7) [247–252]. A fragmentation of the mitochondrial network may also be potentiated by the inhibition of mitophagy [253]. Indeed, overexpression of PINK1 was neuroprotective in *Drosophila* and mouse HD models through increased mitophagy [254]. Wild-type HTT protein may also directly participate in autophagy/mitophagy *via* its interaction with autophagic adaptor, p62 [255], or with the mitophagic protein, BCL2-interacting protein 3 (BNIP3) (Figure 7) [256]. Therefore, mutant HTT may directly disrupt autophagic or mitophagic process *via* these mechanisms.

Furthermore, oxidative stress and neuroinflammation are other common pathogenic factors in HD [257]. A number of ARE-containing genes are found to be induced in human HD brain [258], suggesting that NFE2L2 activation may be involved in the pathogenesis. In animal HD models with mitochondrial complex II inhibition, overexpression of NFE2L2 exerts a neuroprotective effect [259]. While the expression of NFE2L2 protein did not alter in a HD model, the expression of the NFE2L2 modulators, KEAP1 and p62, were found to be reduced, and thus, this could affect NFE2L2 activity [260]. Moreover, cotransfection of NFE2L2 with mutant HTT in primary striatal neurons reduced the half-life of mutant HTT and improved cell viability [261]. In fact, activation of NFE2L2 is protective against mutant HTT-induced toxicity [262, 263], highlighting the potential of NFE2L2 induction for HD patients.

### 7.5. Friedreich's Ataxia (FA)

Friedreich's ataxia is the most prevalent autosomal recessive spinocerebellar disorder that affects approximately one in 50,000 Caucasians [264]. It is characterized by progressive neuro- and cardiodegeneration and mitochondrial iron accumulation [264, 265]. The disorder is predominantly caused by a GAA repeat expansion in the first intron of the *FRDA* gene that results in a marked reduction in the expression of the encoded protein, frataxin [266, 267]. Approximately 2% of the remainder FA cases are due to point mutations in the *FRDA* gene [264].

**Figure 8:**
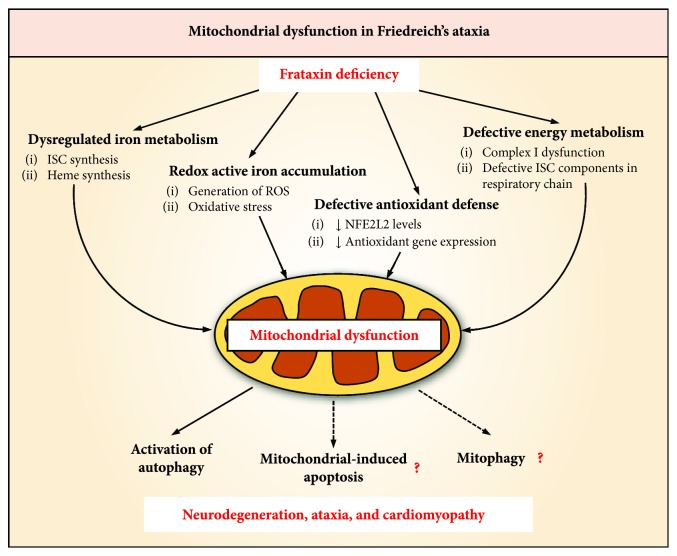
Effect of frataxin deficiency on mitochondrial dysfunction and the pathogenesis of Friedreich's ataxia (FA). It has been well established that frataxin deficiency leads to the dysregulation of mitochondrial iron metabolism that affects the iron-sulfur cluster (ISC) and heme biosynthesis. Notably, there is the abnormal accumulation of redox active iron in the mitochondria that exacerbates ROS generation, which is further potentiated by the defect in antioxidant defense, as evident by the decrease in NFE2L2 levels and its downstream antioxidant target genes. Furthermore, studies have shown that frataxin deficiency disrupts energy metabolism due to the impairment of the mitochondrial respiratory chain. These pathological features collectively attribute to mitochondrial dysfunction in FA, which can activate autophagy and potentially induce apoptosis and mitophagy. As a result, this leads to the neurodegeneration, ataxia, and cardiomyopathy observed in FA.

The manifestation of FA symptoms is most prominently characterized by progressive neurological disability and fatal dilated cardiomyopathy, as well as a tendency for diabetes mellitus in approximately 10% of FA patients [268, 269]. The pathogenesis of FA is associated with mitochondrial iron accumulation that results in ROS-induced toxicity (Figure 8) [270–272]. As such, iron-chelation therapy has been shown to be beneficial in reducing both neurologic and cardiologic FA pathology, presumably by preventing oxidant-mediated cell death [272–275]. In addition to mitochondrial iron accumulation and oxidative damage, FA patients also exhibit a deficit of ISC enzymes, leading to decreased energy metabolism as evident by complex I dysfunction, as well as perturbed heme synthesis (Figure 8) [270, 276]. This is due to the dysregulation of cellular and mitochondrial iron metabolism upon frataxin deficiency, which disrupts proper utilization of iron and causes mitochondrial dysfunction (Figure 8) [271, 277].

Recent studies utilizing a conditional frataxin knockout mice model of FA have demonstrated that frataxin deficiency leads to pronounced trafficking of iron from the cytosol to the mitochondrion, leading to a cytosolic iron deficiency and mitochondrial iron accumulation in the form of nonprotein-bound, biomineral iron aggregates [270, 272, 278]. Due to the depletion of mitochondrial ferritin in frataxin deficiency [270, 278], these iron aggregates within the redox active mitochondria result in increased protein oxidation and depletion of the cellular antioxidant pool [271]. Paradoxically, despite the apparent oxidative stress, the expression and activity of NFE2L2 is markedly depressed [271], due to a mechanism involving increased KEAP1- and GSK3*β*-mediated NFE2L2 degradation in the cytosol and nucleus, respectively (Figure 8) [271]. As such, the marked decrease in NFE2L2 levels results in the deficient expression of its downstream target genes for antioxidant defense, hence exacerbating oxidative stress (Figure 8) [271]. The defective induction of NFE2L2 despite clear oxidative stress in FA suggests that NFE2L2 may be a potential target for treatment against FA.

Frataxin deficiency has also been associated with autophagy and apoptosis. Frataxin-silenced neuron-like cells undergo apoptosis through the upregulation of p53 and BAX, as well as caspase activation, which suggests the involvement of mitochondrial dysfunction in the pathogenic initiation of apoptosis [279]. Notably, increased autophagic and apoptotic markers in a cardiac mouse model of FA that exhibit frataxin deficiency implicate their role in the observed cardiomyopathy [280]. Therefore, mitochondrial dysfunction is probably responsible for the activation of autophagy, and promoting apoptosis, potentially through the intrinsic pathway involving the mitochondrion (Figure 8). Furthermore, considering the extent of mitochondrial dysfunction in FA, it is possible that dynamic mitochondrial processes, such as mitophagy, are also perturbed (Figure 8). Collectively, the resulting accumulation of redox active iron, oxidative stress, defective antioxidant response, dysfunction in energy metabolism, and activation of autophagy and apoptosis due to frataxin deficiency leads to the neurodegeneration, ataxia, and cardiomyopathy in FA (Figure 8).

### 7.6. Potential Therapies for Degenerative Disorders Targeting Mitochondrial Function

There has been a substantial increase in the interest and generation of potential mitochondrial targeted therapeutics over the past 20 years. Several advancements are considered here as interesting examples relevant to the current review. For disease-specific analysis of mitochondrial targeted therapeutics, the reader is encouraged to examine the following comprehensive reviews [281–283].

Mitochondrial dysfunction and damage induced by ROS play a critical role in the pathogenesis of many degenerative diseases [284]. Therefore, NFE2L2 and its signaling pathway have become a major therapeutic target for the treatment of diseases such as AD, PD, ALS, HD, and FA, which focuses on improving mitochondrial bioenergetics and function through the alleviation of oxidative stress and the activation of antioxidant defense [34, 115, 285].

In a study using cellular models of AD, the activation of the NFE2L2 signaling pathway by the potent free radical scavenger, 3H-1,2-dithiole-3-thione, was able to reduce A*β* levels and attenuate ROS generation, which partially rescued mitochondrial membrane potential [115]. In PD models, NFE2L2 inducers are able to restore mitochondrial membrane potential in PINK1-deficient cells and rescue dopamine-induced toxicity [184]. Previous studies on FA have assessed the effectiveness of promoting NFE2L2 levels in the rescue of oxidative stress-induced mitochondrial impairments [286]. One particular study has shown that the NFE2L2-inducer, omaveloxolone, was able to restore complex I activity and protect against oxidative stress in neuronal mouse models of FA, as well as in fibroblasts from FA patients [286]. The mitochondrial membrane potential was also maintained upon incubation of cells with the NFE2L2 inducer, which suggests its potential in improving mitochondrial function in addition to its effect on oxidative stress [286]. As a further example of defense against neural oxidative stress, studies have shown that pretreatment with NFE2L2-inducing agents, sulforaphane, or carnosic acid, was able to induce the NFE2L2 pathway and protect cortical mitochondria from the effects of the neurotoxic lipid peroxidation by-product, 4-hydroxynonenal [287]. Moreover, NFE2L2 has been suggested to also influence mitochondrial activity by affecting the availability of substrates such as NADH and FADH2 for mitochondrial respiration [34, 184]. Hence, the pharmacological activation of NFE2L2 could potentially rescue OXPHOS activity and mitochondrial bioenergetics in disease states.

Alternatively, melatonin is an interesting mitochondria-targeted antioxidant that has been recently explored for the treatment of AD and PD [288–290]. Evidences suggest that the mechanism of neuroprotection of melatonin involved increasing NFE2L2 expression and activation of the NFE2L2/ARE pathway [288–293]. In addition to activating the NFE2L2 pathway, previous studies in mice subjected to irradiation-induced neurodegeneration have also shown that pretreatment of melatonin accumulates in mitochondria and was able to promote PINK1 mitochondrial accumulation that alters mitochondrial dynamics and prevents loss of mitophagic progression [288]. Overall, melatonin pretreatment was able to increase mitochondrial respiratory chain activity and enhance cognitive performance in these animals [288]. In fact, there is evidence to suggest that NFE2L2 can regulate PINK1 expression due to the presence of ARE in the promoter regions of the *PINK1* gene [294, 295]. Hence, NFE2L2 may have a role in mediating mitophagy, and the upregulation of NFE2L2 could potentially restore mitochondrial homeostasis in PD [288].

Additionally, triterpenoids are antioxidants that activate the NFE2L2 signaling pathway to inhibit oxidative stress and were found in a number of studies to have neuroprotective effects that could improve behavioral phenotype in mouse models of AD [296]. In relation to mitochondria, the triterpenoid, asiatic acid, reportedly protects neurons from cell death by preventing mitochondria-dependent apoptosis in a cellular model of AD [297]. Another study also demonstrated that asiatic acid blocked the translocation of *α*-synuclein into mitochondria, thereby protecting it against oxidative stress and apoptosis [298]. Asiatic acid also prevented the *α*-synuclein-induced decrease in mitochondrial membrane potential in a *Drosophila* model of PD [298].

Impairment of the NFE2L2 signaling pathway and mitochondrial dysfunction is evident in the pathogenesis of degenerative diseases, yet the development of drugs that exploit the targeting of mitochondria through the activation of NFE2L2 is only in its infancy. Recent studies have already begun to demonstrate the effect of this relationship to rescue mitochondrial function in neurodegenerative diseases. Hence, the combined effect of ameliorating oxidative stress and mitochondrial dysfunction would be a novel approach in future drug design for the treatment of various neurodegenerative diseases.

Another avenue in the development of new therapeutics involves the targeted chelation of cytosolic and/or mitochondrial iron, as it is known to play a significant role in potentiating oxidative stress and ROS generation for many degenerative diseases in addition to a defect in mitochondrial respiration [299–304].

Previous studies on AD have shown that the sequestration of iron by FtMt have neuroprotective effects in cell models, which prevented neuronal cell damage induced by A*β* [128]. Similarly, the regulation of FtMt helps maintain mitochondrial and neuronal iron homeostasis, in which its overexpression was shown in a mouse model of PD to inhibit mitochondrial damage and reduce ROS production, thus having neuronal protection [305]. These studies indicate the importance of regulating mitochondrial iron levels, especially in iron overload conditions. Studies have also examined the use of metal chelation, such as the metal-binding agent, PBT2, that has been in clinical trials for the treatment of AD and HD, which demonstrated the potential for binding iron, copper, and zinc in the brain and reduce amyloid plaque formation with signs of cognitive improvement [306]. The design of chelators with specificity for mitochondrial iron and other metals could increase the effectiveness of iron chelation therapy for neurodegenerative diseases. Other studies have also reported that intracellular oxidative stress enhances HO-1 activity that leads to the accumulation of iron in the mitochondria of astrocytes in AD and PD brains [307]. This deposition of mitochondrial iron in glial cells increases the risk of neighboring neurons to further oxidative damage [307, 308]. These findings give rise to the prospect of iron chelation therapy, especially in the targeting of mitochondrial iron, as a new neuroprotective strategy for AD and PD.

In FA, mitochondrial iron loading is well-characterized in the heart of a mouse cardiac model of FA, which resulted in severe defects in mitochondrial function [272, 280, 309, 310]. In fact, Mӧssbauer spectroscopic analysis and transmission electron microscopy demonstrate that iron appears as a nonferritin, high spin form of ferric iron that exists without a protein shell that prevents against ROS-mediated oxidative damage [278]. As such, this precipitation of mitochondrial iron potentiates redox stress, which warrants for its targeted removal. A specialized group of low molecular weight, lipophilic ligands of the pyridoxal isonicotinoyl hydrazone (PIH) class [311] has been examined to target this mitochondrial iron loading [312]. In terms of mechanism, PIH could permeate biological membranes, including the mitochondrion, and effectively chelate mitochondrial iron accumulation after mitochondrial heme synthesis was inhibited using succinylacetone, as demonstrated in reticulocytes [313]. Further studies demonstrated that PIH and several of its analogs [314] effectively removed mitochondrial iron [312] and inhibited oxidative stress [315]. Moreover, these latter agents were markedly superior to desferrioxamine (DFO) [314], a chelator used for treating iron overload disease [316]. Interestingly, in the mouse cardiac model of FA, the combination of PIH and DFO prevented iron loading in the heart and reduced cardiac hypertrophy but did not rescue the defective iron metabolism caused by the loss of frataxin [272]. Considering that the spinal cord and dorsal root ganglia are highly vulnerable to frataxin deficiency [317, 318], cellular and mitochondrial iron dysregulation could contribute to the pathophysiology of these tissues. As such, it is necessary to consider the design of iron chelators that targets these pathogenic regions.

The use of iron chelators in targeting mitochondrial iron as a therapeutic strategy for degenerative diseases deserves further investigation. The combination of antioxidants and iron chelators could potentially be an effect approach to ameliorate oxidative stress and boost mitochondrial function, while also eliminating harmful iron that would otherwise potentiate redox damage.

## 8. Conclusions

The mitochondrion emerges as a central hub that orchestrates cellular antioxidant defense, energy production, and apoptosis. The regulation of NFE2L2 has been shown to play an essential role in antioxidant defense, and the role of this key protein in mitochondrial homeostasis has only been recently elucidated, linking antioxidant defense to neuronal mitochondrial trafficking. This includes recent evidence of the direct interaction of NFE2L2 with this organelle.

The exquisite dependence of high energy–demanding cells, such as neurons and cardiomyocytes, on mitochondria for energy production means that mitochondrial dysfunction can lead to their demise. This is exemplified by the number of deleterious neurodegenerative diseases, such as AD, PD, ALS, HD, and FA, where perturbation of mitochondrial function is an essential component of their pathogenesis. Therefore, the maintenance of mitochondrial homeostasis is a crucial factor and a potential therapeutic target to treat these diseases. Our understanding of mitochondrial homeostasis and metabolism is also broadened by the discovery of novel mutations causing these neurodegenerative diseases, with an appropriate example being the identification of the role of frataxin in Friedreich's ataxia.

Considering the key role of the mitochondrion in this array of degenerative diseases, therapeutic strategies targeting this organelle has been a focus of the increasing body of research. The additional linkage of NFE2L2 with mitochondria may lead to the convergence of previously considered disparate avenues of treatment that could result in exciting and innovative therapeutic advances.
